# M2b macrophages stimulate lymphangiogenesis to reduce myocardial fibrosis after myocardial ischaemia/reperfusion injury

**DOI:** 10.1080/13880209.2022.2033798

**Published:** 2022-02-21

**Authors:** Cuiping Wang, Yuan Yue, Suiqing Huang, Keke Wang, Xiao Yang, Jiantao Chen, Jiaxing Huang, Zhongkai Wu

**Affiliations:** a Department of Cardiothoracic ICU, The First Affiliated Hospital of Sun Yat-Sen University, Guangzhou, PR China; b Key Laboratory on Assisted Circulation, Ministry of Health, Guangzhou, PR China; c Department of Cardiac Surgery, The First Affiliated Hospital of Sun Yat-Sen University, Guangzhou, PR China; d Department of Laboratory Medicine, Guangzhou First People's Hospital, School of Medicine, South China University of Technology, Guangzhou, PR China

**Keywords:** Cardiac fibrosis, VEGFC, VEGF receptor 3

## Abstract

**Context:**

Therapeutic lymphangiogenesis is a new treatment for cardiovascular diseases. Our previous study showed M2b macrophages can alleviate myocardial ischaemia/reperfusion injury (MI/RI). However, the relation between M2b macrophages and lymphangiogenesis is not clear.

**Objective:**

To investigate the effects of M2b macrophages on lymphangiogenesis after MI/RI.

**Materials and methods:**

Forty male Sprague-Dawley (SD) rats were randomized into Sham operation group (control, *n* = 8), MI/RI group (*n* = 16) and M2b macrophage transplantation group (*n* = 16). M2b macrophages (1 × 10^6^) in 100 μL of normal saline or the same volume of vehicle was injected into the cardiac ischaemic zone. Two weeks later, echocardiography and lymphatic counts were performed, and the extent of myocardial fibrosis and the expression of vascular endothelial growth factor C (VEGFC) and VEGF receptor 3 (VEGFR3) were determined. *In vitro,* lymphatic endothelial cells (LECs) were cultured with M2b macrophages for 6–24 h, and the proliferation, migration and tube formation of the LECs were assessed.

**Results:**

*In vivo,* M2b macrophage transplantation increased the level of lymphangiogenesis 2.11-fold, reduced 4.42% fibrosis, improved 18.65% left ventricular ejection fraction (LVEF) and upregulated the expressions of VEGFC and VEGFR3. *In vitro,* M2b macrophage increased the proliferation, migration, tube formation and VEGFC expression of LECs. M2b macrophage supernatant upregulated VEGFR3 expression of LECs.

**Discussion and Conclusions:**

Our study shows that M2b macrophages can promote lymphangiogenesis to reduce myocardial fibrosis and improve heart function, suggesting the possible use of M2b macrophage for myocardial protection therapy.

## Introduction

Cardiac lymphatics drain tissue fluid to maintain a steady-state interstitial fluid equilibrium in the heart, and lymphatic function is critical for maintaining cardiac function. The role of cardiac lymphatics in normal physiology and pathological conditions has not been fully studied, largely due to technical challenges in visualizing cardiac lymph flow and lymphatic vessels (Choi et al. 2012). The identification of specific lymphatic endothelial cell (LEC) regulators and markers, such as vascular endothelial growth factor C (VEGFC) (Joukov et al. 1996), VEGF receptor 3 (VEGFR3) (Lohela et al. 2009), lymphatic vessel endothelial hyaluronan receptor 1 (LYVE1) (Banerji et al. 1999), podoplanin (Breiteneder-Geleff et al. 1999) and prosperohomeobox 1 (PROX1) (Wigle and Oliver 1999) has facilitated studies of the cardiac lymphatic system. Studies have indicated that exogenous VEGFC stimulates cardiac lymphangiogenesis to reduce the extent of myocardial edoema and fibrosis, and improve heart function (Henri et al. 2016). Therapeutic lymphangiogenesis may be a promising approach for the treatment of cardiovascular diseases (Huang et al. 2017; Vuorio et al. 2017).

Macrophages are one of the most important cell types that participate in lymphangiogenesis (Watari et al. 2008; Yamashita et al. 2009). Our previous study showed that the transplantation of M2b macrophages into the heart can significantly attenuate myocardial ischaemia/reperfusion injury (MI/RI) during the early period (Yue et al. 2017). However, it has not been reported whether M2b macrophages, as regulatory macrophages, can affect lymphangiogenesis.

This study determines if M2b macrophages can promote cardiac lymphangiogenesis, and thus inhibit myocardial fibrosis and improve cardiac function in the late stage of MI/RI. The role of the VEGFC/VEGFR3 signalling pathway in this process was also investigated to better understand the mechanism by which M2b macrophages attenuate MI/RI injury.

## Materials and methods

### Animals

A total of fifity adult, male Sprague-Dawley (SD) rats (weight 180–220 g) were obtained from the Laboratory Animal Centre of Guangzhou University of Chinese Medicine. Forty rats were randomly assigned to three groups: (1) Sham operation group (control, *n* = 8); (2) MI/RI group (I/R + Vehicle, *n* = 16); (3) M2b macrophage transplantation group (I/R + M2b, *n* = 16). Because of the homogeneity of the sham operation group, only eight rats were used to meet the ‘3R rule’ (replacement, reduction and refinement). The other ten rats were killed to harvest macrophages. All animals were maintained under constant temperature (22 ± 2 °C), humidity (45 ± 5%), and a 12 h day/night cycle, and were allowed free access to food and water. All animal procedures were approved by the Institutional Animal Care Committee of Sun Yat-Sen University, and were performed in accordance with National Institutes of Health (NIH) guidelines (The National Academies Collection: Reports funded by National Institutes of Health, 2011).

### Cells

M2b macrophages were polarized from bone marrow-derived macrophages (BMDMs) harvested from the rats. In brief, the rats were killed by dislocation of the cervical vertebrae, and bone marrow was collected from the tibias and femurs. The bone marrow was washed with complete Roswell Park Memorial Institute (RPMI) 1640 medium (Gibco, Grand Island, NY) and centrifuged at 500 *g* for 5 min. The resulting cells were collected and cultured in flasks (Corning, NY) at 37 °C in a 5% CO_2_ incubator in RPMI 1640 for the first 3 d, and then in Dulbecco’s Modified Eagle’s medium (DMEM; Gibco) for the next 3 d to allow formation of mature BMDMs. Both RPMI 1640 and DMEM were supplemented with 10% foetal bovine serum (FBS) (Gibco) and 20 ng/mL macrophage colony-stimulating factor (PeproTech, Rocky Hill, NJ). On day 6, the BMDMs were differentiated into M2b macrophages by treatment with lipopolysaccharide (LPS) (100 ng/mL, Sigma Aldrich, St. Louis, MO) and IgG (50 μg/mL, Sigma-Aldrich) for 48 h. M2b macrophages were identified by flow cytometry and quantitative real-time PCR (qRT-PCR) as described previously (Yue et al. 2017).

LECs derived from the thoracic ducts of normal adult SD rats were purchased from iCell Bioscience Inc. (Shanghai, China), and were identified by immunofluorescence staining of VEGFR3 and lymphatic vessel endothelial hyaluronan receptor 1 (LYVE1).

### Rat model and M2b macrophage transplantation

Polarized M2b macrophages were washed in once with PBS and resuspended in normal saline. Normal saline was also used as the vehicle in for transplantation. After anaesthesia with 2% nembutal sodium (50 mg/kg), endotracheal intubation was performed on the rats and artificial ventilation (4–5 mL tidal volume, 90 breaths/min) was begun with a rodent ventilator. A left thoracotomy through the fourth intercostal space was performed and the left anterior descending (LAD) coronary artery was occluded for 45 min with an 8-0 Prolene suture. The suture was removed after 45 min, and 1 min later the ischaemic area was identified. Either 1 × 10^6^ M2b macrophages in 100 μL of normal saline (I/R + M2b group) or the same volume of normal saline (I/R + Vehicle group) was injected into the ischaemic border zone at five sites. Rats in the sham operation group (Control group) underwent the same operation without ligation of the LAD coronary artery.

After the procedures, gas was squeezed from the chest and the intercostal muscles and skin were sutured. The rats were placed on a heated blanket and fed in their cages after awakening. Rats that exhibited severe arrhythmia, cardiac arrest or respiratory failure during the procedure were excluded from the study, and one rat was excluded from the I/R + Vehicle and one from the I/R + M2b group. No rats were excluded from the Control group. Two weeks after the procedure the rats were killed by exsanguination after anaesthesia with 2% nembutal sodium. Blood was collected from the caudal vein and the hearts were harvested.

### Echocardiography

Rats were anaesthetized with 2% nembutal sodium, and transthoracic echocardiography was performed (Visual Sonics system, Toronto, Ontario, Canada). M-mode and 2-dimensional echocardiography were performed to assess cardiac parameters, including the left ventricular (LV) end-diastolic dimension, wall thickness, LV fractional shortening (LVFS) and ejection fraction (EF).

### Detection of myocardial fibrosis

Hearts were fixed in 4% paraformaldehyde and transected along the area of the LV infarction. Paraffin-embedded 5 μm sections were obtained, and stained with Masson trichrome for identification of collagen. The ratio of collagen fibres in the infarct area to the total area was determined. The slides were observed under a compound microscope (Olympus Optical Co., Ltd., Tokyo, Japan), and the degree of fibrosis was determined as the percentage of the blue-stained area compared to the total area of the left ventricle using Image J software (National Institutes of Health, Bethesda, MA).

### Histological analysis

Immunohistochemical (IHC) staining for lymphatic tissue was performed using a primary antibody against LYVE1 (Novus Biologicals), and a horse radish peroxidase (HRP)-conjugated secondary antibody (Invitrogen, Shanghai, China). Sections were randomly selected from the ischaemic myocardium, and five areas were randomly selected from each section. The number of lymphatic vessels in the fibrotic section was calculated by two individuals blinded to the experimental group. Only vessel structures were assessed and individual endothelial cells were not counted. If one vessel was divided into two branches, it was counted as two vessels. If two vessels were adjacent and partly overlapping, they were counted as two vessels.

### Western blotting

Protein was extracted from the hearts using the AllPrep DNA/RNA/Protein Mini Kit (QIAGEN, Duesseldorf, Germany) according to the manufacturer’s instructions. Protein extracts (40 µg) were separated by 10–12% sodium dodecyl sulphate-polyacrylamide gel electrophoresis (SDS-PAGE). The blots were probed with primary antibodies against VEGFC or VEGFR3 (Abcam, Cambridge, UK). HRP-conjugated anti-rabbit or anti-rat IgG (Southern Biotech, Birmingham, AL) were used as the secondary antibodies.

### Cell counting kit-8 (CCK-8) assay

Rat LECs were seeded in 96-well plates (Corning) at a cell density of 1 × 10^4^ cells per well, and were allowed to attach overnight. The cells were treated with DMEM (control group) or M2b macrophage supernatant (M2b group) for 24 h. Cell viability was then measured using the CCK-8 assay (Beyotime Biotechnology, Shanghai, China). The absorbance value at 450 nm was read using a Sunrise Microplate Reader (Tecan Group Ltd., Männedorf, Switzerland).

### Transwell migration assay

The Transwell migration assay was performed using 24-well, 8 μm pore membranes according to the manufacturer’s protocol (Corning, NY). A total of 5 × 10^4^ LECs were seeded in the upper compartment in 200 μL of DMEM without serum. In the lower chambers, 1 mL of M2b macrophage supernatant (M2b group) or DMEM (control group) was added. After incubation for 24 h at 37 °C in a 5% CO_2_ incubator, the cells remaining on the upper surface of the membrane were removed with cotton swabs, and the number of cells that reached the lower chamber was counted to determine cell migration. The migrated cells were fixed with 4% paraformaldehyde, and stained with 0.4% crystal violet solution. The cells that passed through the filter were photographed under a compound microscope (Leica Application Suite). The number of LECs in the filter was quantified by counting migrated cells in ten fields of vision from three independent experiments using Image J software.

### Tube formation assay

A tube formation assay was performed by pipetting 300 μL of Matrigel (BD Biosciences, Bedford, MA) into each well of a 24-well plate. The Matrigel was then polymerized for 30 min at 37 °C. LECs (5 × 10^5^) were resuspended in 500 μL of DMEM (control group) or 500 μL of M2b macrophage supernatant (M2b group), and were added to each well. The cultures were incubated at 37 °C in a 5% CO_2_ atmosphere for 6 and 10 h. Images were captured at each time point using a bright-field ZEISS Axio Observer Z1 microscope (Carl Zeiss Microscopy, LLC, Thornwood, NY). Tube parameters were calculated using Image J software.

### Quantitative real-time PCR (qRT-PCR)

RNA was extracted from co-cultured LECs and M2b macrophages using Trizol (Invitrogen, Shanghai, China), according to the manufacturer’s instructions. RNA was reverse-transcribed into cDNA with a RevertAid First Strand cDNA Synthesis Kit (Thermo Fisher Scientific, Waltham, MA). Then, qRT-PCR was performed on a Light Cycler 480 system (Roche Applied Science, Mannheim, Germany) using Maxima SYBR Green qPCR Master Mix (Thermo Fisher Scientific), according to the manufacturer’s instructions. The comparative threshold cycle (CT) value for Tubulin was used to normalize the loading variations in the assays. The primer sequences for the target genes were: VEGFC forward: CTACAGATGTGGGGGTTGCT, reverse: GCTGCCTGACACTGTGGTAA; VEGFR3 forward: CTCCAACTTCTTGCGTGTCA, reverse: ACAAGGTCCTCCATGGTCAG; Tubulin forward: AGCCATGTACGTAGCCATCC, reverse: CTCTCAGCTGTGGTGGTGAA.

### Immunofluorescence

LECs were fixed in 4% paraformaldehyde and permeabilized with 0.2% Triton X-100 at room temperature. After blocking with donkey serum in PBS containing 0.2% Triton X-100 for 0.5 h, the LECs were incubated with primary antibodies against VEGFR3 (Abcam) and LYVE1 (Novus Biologicals) for 1 h at room temperature. After washing three times with PBS, the LECs were incubated for 1 h with a secondary antibody at room temperature. The slides were washed three times with PBS, mounted with Fuoroshield mounting medium containing 4′,6-diamidino-2-phenylindole (DAPI: Sigma), and sealed with nail polish. Slides were observed by confocal microscopy (Carl Zeiss Microscope).

### Statistical analysis

Data were presented as mean ± standard error of the mean (SEM). Statistical analyses were performed using GraphPad Prism software version X (La Jolla, CA) and SPSS version 16.0 software (SPSS Inc., Armonk, NY). Data between two groups were compared with Student’s *t*-test. Comparisons among groups were assessed by a one-way analysis of variance (ANOVA). In all analyses, a value of *p* < 0.05 was considered statistically significant.

## Results

### M2b macrophage transplantation improves cardiac function and inhibits fibrosis

**Figure 1.**
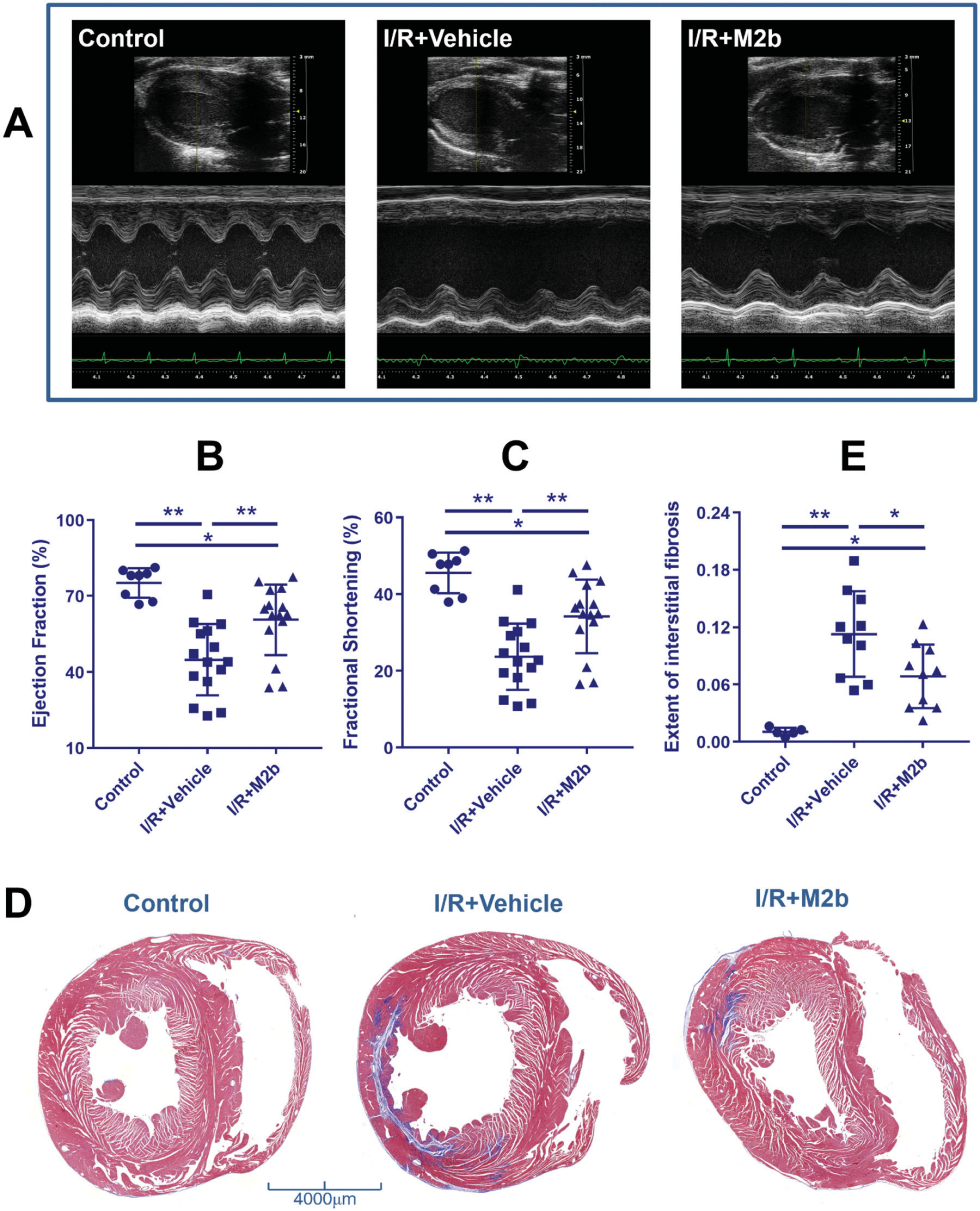
M2b macrophage transplantation improves cardiac function and inhibits fibrosis after MI/RI.

The echocardiographic parameters of the three groups are summarized in Table 1. Rats in the I/R + Vehicle group and the I/R + M2b group exhibited significantly worse cardiac function than those in the Control group with respect to LVEF and LVFS (all, *p* < 0.05). The parameters LVEF and LVFS were significantly higher in the I/R + M2b group than in the I/R + Vehicle group (both, *p* < 0.05) (Figure 1(A–C)). The I/R + Vehicle group and the I/R + M2b group had significantly lower interventricular septum (IVS) and LV posterior wall (LVPW), and greater LV internal diameter (LVID) than the Control group (all, *p* < 0.05). In addition, the end-diastolic and end-systolic LVID were significantly smaller in the I/R + M2b group than in the I/R + Vehicle group (both, *p* < 0.05) (Table 1).

**Table 1.** Echocardiographic parameters.

Variables	Control (*n* = 8)	I/R + Vehicle (*n* = 15)	I/R + M2b (*n* = 15)
HR (beats/min)	376.35 ± 73.07	361.69 ± 69.15	391.76 ± 113.46
IVS; d (mm)	1.92 ± 0.31	1.53 ± 0.46*	1.42 ± 0.36*
IVS; s (mm)	2.94 ± 0.53	1.91 ± 0.65*	1.99 ± 0.52*
LVID; d (mm)	7.16 ± 0.50	7.91 ± 0.86*	7.11 ± 0.55^&^
LVID; s (mm)	3.90 ± 0.47	6.07 ± 1.13*	4.54 ± 0.77^*&^
LVPW; d (mm)	2.11 ± 0.47	1.85 ± 0.32	1.76 ± 0.29*
LVPW; s (mm)	2.98 ± 0.36	2.43 ± 0.48*	2.81 ± 0.50
LVEF (%)	75.16 ± 5.86	44.87 ± 14.06*	63.52 ± 12.48^*&^
LVFS (%)	45.52 ± 5.30	23.70 ± 8.64*	36.26 ± 8.92^*&^

Data were expressed as the means ± SEM. IVS; d: interventricular septum (diastolic); IVS; s: interventricular septum (systolic); LVID; d: end-diastolic left ventricular internal diameter; LVID; s: end-systolic left ventricular internal diameter; LVPW; d: left ventricular posterior wall (diastolic); LVPW; s: left ventricular posterior wall (systolic); LVEF: ejection fraction of the left ventricle; LVFS: fractional shortening of the left ventricle

*p < 0.05 *vs.* Control.

^&^*p* < 0.05 *vs.* I/R + Vehicle.

Cardiac fibrosis was evaluated by Masson trichrome staining of collagen fibres, and there was a significant increase in collagen after MI/RI (*p* < 0.01, one-way ANOVA), mainly in the infarct area. Collagen fibres were significantly reduced 4.42% in the I/R + M2b group as compared to the I/R + Vehicle group (*p* < 0.05) (Figure 1(D,E)).

### M2b macrophage transplantation promotes cardiac lymphangiogenesis

**Figure 2.**
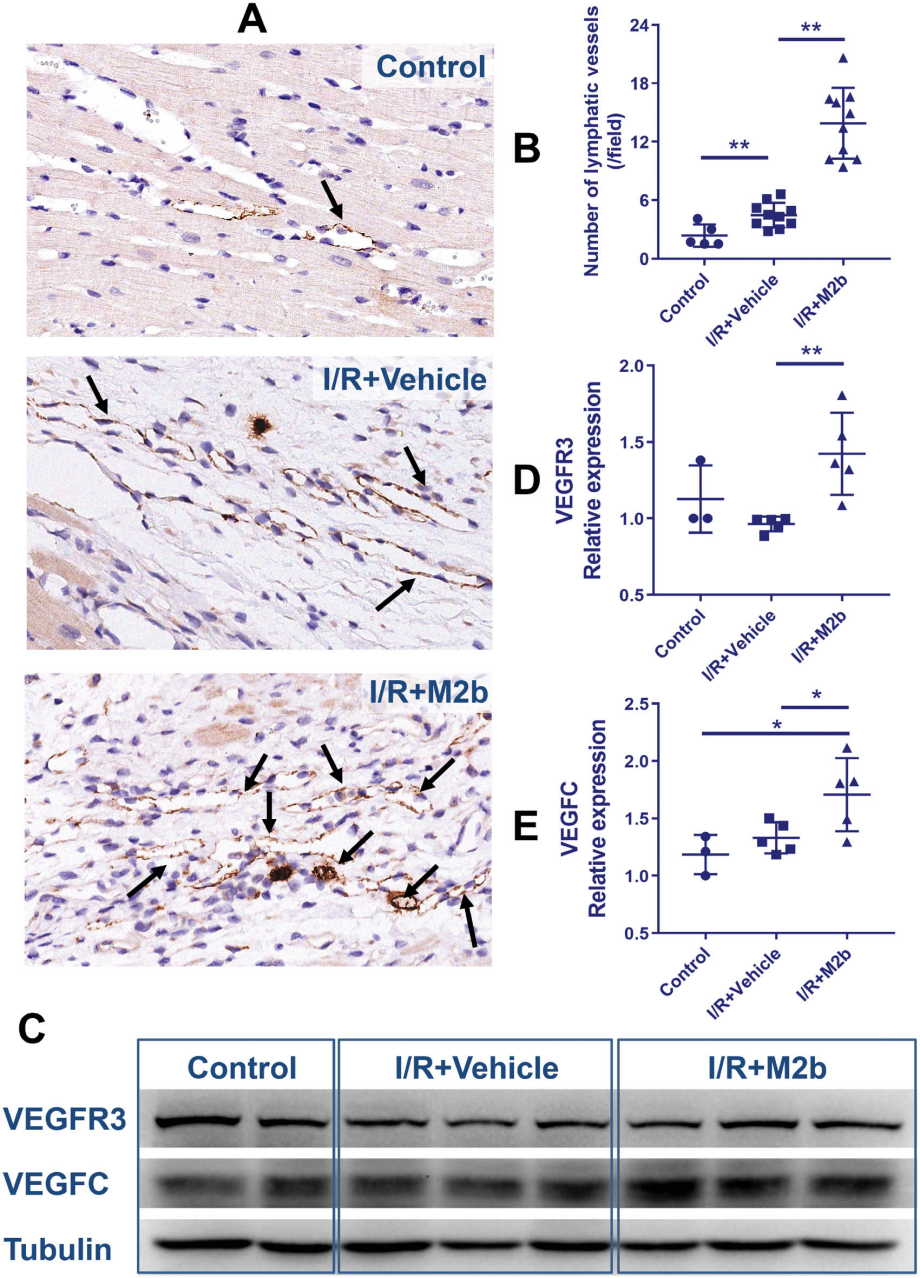
M2b macrophage transplantation promotes cardiac lymphangiogenesis after MI/RI.

There were more lymph vessels (as indicated by staining for LYVE1) in the I/R + M2b group and the I/R + Vehicle group as compared to the Control group (both, *p* < 0.01). Compared to the I/R + Vehicle group, the mean number of lymphatic vessels was 2.11-fold higher in the infarct area of the I/R + M2b group (*p* < 0.01) (Figure 2(A,B)).

VEGFC and VEGFR3 are important markers of lymphangiogenesis. The number of lymphatic vessels and levels of VEGFR3 and VEGFC of the three groups are shown in Figure 2. As detected by Western blotting, the expression of VEGFR3 was significantly higher in the I/R + M2b group than in the I/R + Vehicle group (*p* < 0.01) (Figure 2(C,D)). However, there was no difference in VEGFR3 staining between the I/R + Vehicle group and the Control group (*p* = 0.105) (Figure 2(D)). The expression of VEGFC in the myocardium was similar to that of VEGFR3, and the VEGFC level was significantly higher in the I/R + M2b group than in the other two groups (both, *p* < 0.05) (Figure 2(C,E)), and there was also a significant difference between the I/R + Vehicle and Control group (*p* = 0.015).

### M2b macrophages accelerate the migration and proliferation of LECs

**Figure 3.**
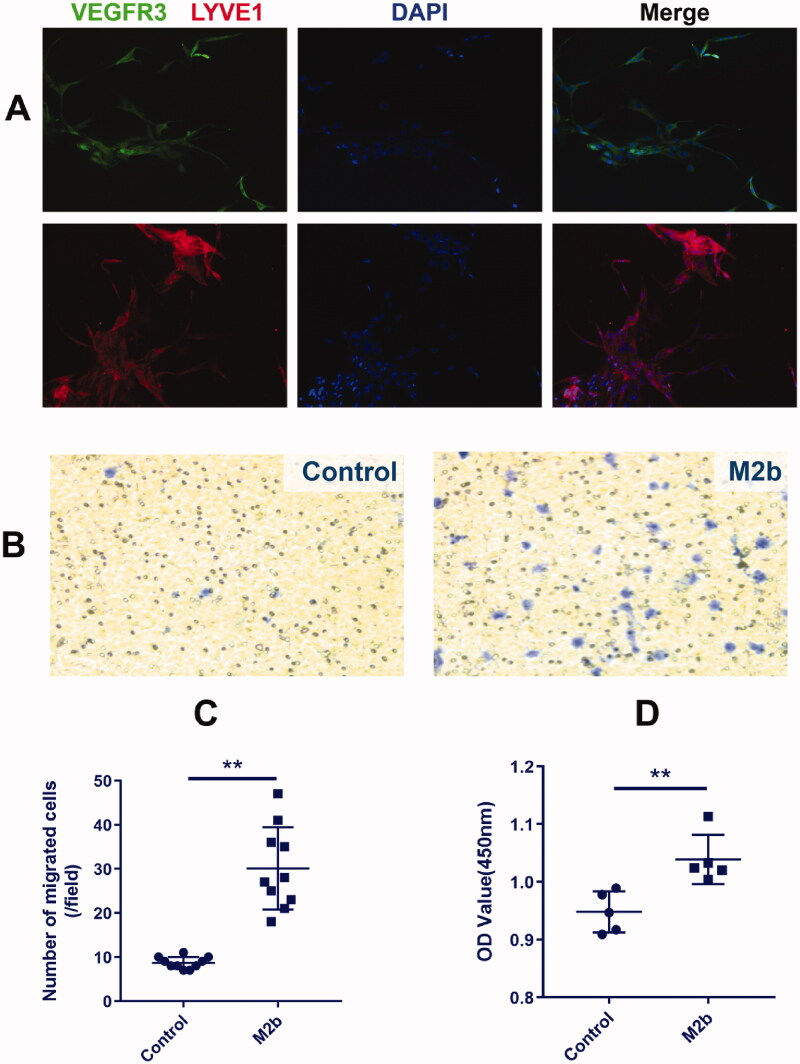
M2b macrophages accelerate the migration and proliferation of LECs.

Immunofluorescence staining of VEGFR3 and LYVE1 was used to identify LECs, and the results showed that the positive rate was 100% (Figure 3(A)). As shown in Figure 3(B,C), the migratory capacity of LECs cultured with M2b macrophage supernatant was significantly enhanced (Transwell migration assay; *p* < 0.01). The proliferation of LECs cultured with M2b supernatant was also increased compared to those cultured with DMEM (Control) (CCK-8 assay; *p* < 0.01) (Figure 3(D)).

### M2b macrophages promote tube formation and VEGFR3 expression of LECs

**Figure 4.**
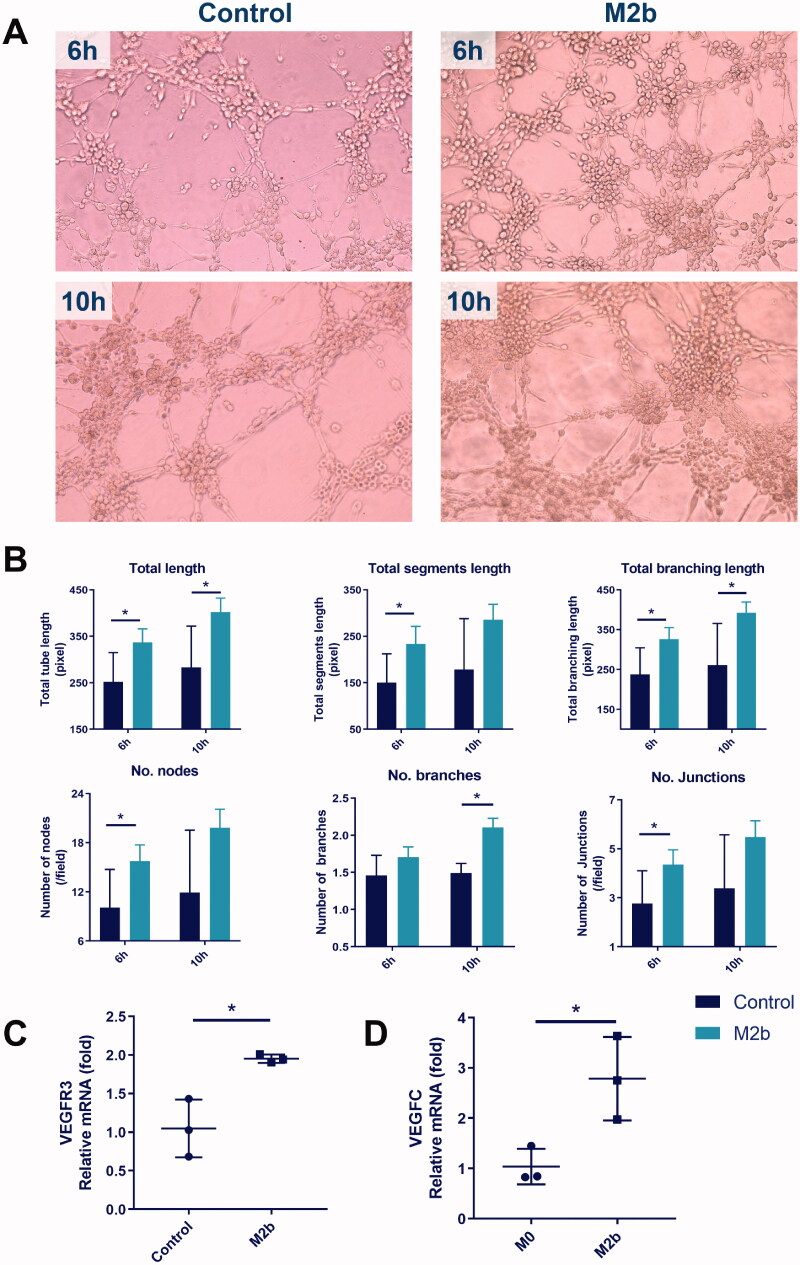
M2b macrophages promote tube formation and VEGFR3 expression of LECs.

The effectiveness of M2b macrophage supernatant in increasing the tube formation of LECs after 6 and 10 h of treatment, as assessed by the total tube length, total segment length, total branching length, number of nodes, number of branches and number of junctions, is illustrated in Figure 4(A,B). As indicated, M2b macrophage supernatant significantly increased the total length and total branching length after 6 and 10 h of treatment (both, *p* < 0.05). M2b macrophage supernatant significantly increased total segment length, and the numbers of nodes and junctions after 6 h of treatment (all, *p* < 0.05). The number of branches in the M2b group was only significantly larger than the control group after 10 h of treatment (*p* < 0.05).

In addition, the mRNA expression of VEGFR3 in LECs was significantly increased by culture with M2b macrophage supernatant (Figure 4(C)) as compared to culture in DMEM (Control) (*p* < 0.05). Finally, VEGFC expression was higher in M2b macrophages than in undifferentiated (M0) macrophages (Figure 4(D)) (*p* < 0.05).

## Discussion

This study was designed to investigate whether M2b macrophages affect lymphangiogenesis to inhibit myocardial fibrosis in the late stage of MI/RI, and to explore the potential mechanisms. *In vivo*, M2b macrophages promoted lymphangiogenesis to reduce myocardial fibrosis, improve heart function, and reduce cardiac remodelling. VEGFC and VEGFR3 expression were upregulated after M2b macrophage transplantation. *In vitro* experiments showed that M2b macrophages stimulate the proliferation, migration, and tube formation of LECs. In addition, the expression of VEGFC was upregulated in M2b macrophages compared to M0 macrophages. The expression of VEGFR3 was also upregulated in LECs cultured with M2b macrophage supernatant compared to those cultured with DMEM.

Macrophages are innate immune cells that are widely distributed in organs and tissues, and they play essential roles in a variety of physiological and pathological processes, such as organ development, host defence, acute and chronic inflammation, and tissue homeostasis and remodelling (Martinez et al. 2008). Typically, macrophages can be divided into two subclasses; classically activated macrophages (M1) and alternatively activated macrophages (M2) (Mills et al. 2000; Martinez and Gordon 2014; Shapouri-Moghaddam et al. 2018). M2 macrophages can be further polarized to M2a, M2b and M2c macrophages (Chanmee et al. 2014; Wang LX et al. 2019). M1 macrophages, which are induced by LPS and interferon (IFN)-γ, produce high levels of pro-inflammatory cytokines (interleukin [IL]-1β, IL-6, IL-12 and IL-23), mediate tissue damage and impair wound healing (Martinez and Gordon 2014). M2a and M2c macrophages secrete high levels of pro-fibrotic transforming growth factor (TGF)-β, inducing tissue fibrosis (Wang Y et al. 2015, 2017). M2b macrophages, also known as regulatory macrophages, express high levels of CCL1 and LIGHT. M2b macrophages maintain the balance between pro-inflammatory and anti-inflammatory functions, and studies have shown that M2b macrophages can reduce the effects of spinal cord injury and MI/RI (Gensel and Zhang 2015; Yue et al. 2017).

M2 macrophages are highly heterogeneous, which allows them to play different roles in the regulation of cardiac fibrosis in injured hearts. Yang M et al. (2012) used an angiotensin II-induced hypertensive cardiac model, and found that M2 macrophages contributed to cardiac fibrosis. By contrast, a study in a mouse model of prediabetes showed that M2 macrophages attenuated cardiac fibrosis (Urbina and Singla 2014). M2 macrophages are thus able to play different roles with respect to cardiac fibrosis. However, these differences may be related to the misclassification of these cells, as there are at least two different subpopulations of M2 macrophages (M2a and M2b). In a CB2 cannabinoid receptor-deficient ischaemic heart model, activation of the M2a macrophage subpopulation led to adverse myocardial fibrosis (Duerr et al. 2014). A relation between M2b macrophages and cardiac fibrosis has not been reported. In a mouse model of hepatic fibrogenesis induced by carbon tetrachloride (CCL_4_), serum amyloid A (SAA) was found to induce M2b-like macrophage polarization during liver inflammation, resulting in protection of the liver from fibrogenesis (Wang Y et al. 2017). However, the mechanism(s) underlying the anti-fibrotic effects is not clear.

Recently, the cardiac lymphatic system has become an area of active research (Henri et al. 2016; Huang et al. 2017; Vuorio et al. 2017). The lymphatic vessel network of the heart regulates many physiological processes that are important for heart function, such as fluid balance, the transport of extravasated proteins and the trafficking of immune cells (Huang et al. 2017). Impairment of cardiac lymphatic flow leads to excess fluid accumulation and myocardial interstitial fibrosis and subsequent heart dysfunction, and is related to several pathological states including atrial fibrillation (Lupinski 2009), pulmonary artery hypertension (Cui et al. 2001), myocardial ischaemia (Henri et al. 2016; Hartikainen et al. 2017) and valvular heart diseases (Niinimaki et al. 2016). Kong et al. (2006) used a rabbit model and found that obstruction of cardiac lymph flow led to enhanced myocardial collagen synthesis and compromised cardiac function. Another study suggested that although myocardial infarction (MI) induces endogenous cardiac lymphangiogenesis, remodelling and dysfunction of the lymphatics contributes to the development of chronic myocardial edoema and inflammation (Henri et al. 2016). This aggravates cardiac fibrosis and dysfunction, as well as promoting exogenous VEGFC expression, in turn stimulating cardiac lymphangiogenesis to reduce myocardial edoema and fibrosis, and eventually improving heart function. In a phase I/IIa study with 1-year follow-up, gene therapy promoting lymphangiogenesis and angiogenesis increased the myocardial perfusion reserve in patients with refractory angina (Hartikainen et al. 2017). A number of studies have suggested that lymphangiogenic therapy may be a promising approach for the treatment of cardiovascular diseases (Henri et al. 2016; Hartikainen et al. 2017; Huang et al. 2017; Vuorio et al. 2017).

Macrophages produce VEGFC to promote lymphangiogenesis (Watari et al. 2008; Yamashita et al. 2009). The phenotypic heterogeneity of macrophages relates to unique functions specific to the local microenvironment. In a mouse model of renal fibrosis, a VEGFR3 inhibitor down-regulated the expression of VEGFC by M1 macrophages to reduce lymphangiogenesis and ameliorate tubule-interstitial fibrosis. M1 macrophages can cause chronic inflammation-induced lymphangiogenesis, which worsens renal fibrosis (Hwang et al. 2019). Our previous study showed that M2b macrophages can significantly reduce the level of cardiac troponin I (cTnI) and the infarct area during the early period of MI/RI (Yue et al. 2017). Whether M2b macrophages can inhibit long-term cardiac fibrosis after MI/RI has not been studied.

Signalling via VEGFC, VEGFD and VEGFR3 is a central molecular mechanism of lymphangiogenesis (Lohela et al. 2009; Ochsenbein et al. 2016; Yang GH et al. 2017; Sainz-Jaspeado and Claesson-Welsh 2018), and the VEGFC/VEGFR3 pathway is one of the classic pathways that produce lymphatic vessels (Lohela et al. 2009; Hagura et al. 2014; Vaahtomeri et al. 2017). Therefore, our initial hypothesis was that M2b macrophages may produce VEGFC to promote cardiac lymphangiogenesis via the VEGFC/VEGFR3 pathway.

We started our investigation with *in vivo* experiments. A rat MI/RI model was established to evaluate the effects of M2b macrophages in the late stage of MI/RI. The results showed that there were more lymph vessels in the I/R groups than in the Control group, which indicated that MI/RI led to the growth of cardiac lymph vessels. Previous studies have also found lymphangiogenesis after MI (Ishikawa et al. 2007; Henri et al. 2016). Newly formed lymphatics may be involved in the maturation of fibrotic tissue and scar formation through the drainage of excess proteins and fluid. The fluid that accumulates in the cardiac interstitial space after myocardial ischaemia leads to myocardial edoema, which can trigger cardiac fibrosis and a deterioration in heart function. Lymphangiogenesis can drain the interstitial fluid to reduce cardiac edoema and collagen synthesis (Huang et al. 2017). Importantly, we found that injection of M2b macrophages improved cardiac function and alleviated fibrosis. We, therefore, concluded that M2b macrophages promote lymphangiogenesis after MI/RI to inhibit cardiac fibrosis and improve cardiac function.

Then, we determined whether M2b macrophages affect LECs by culturing LECs with M2b macrophage supernatant *in vitro*. The results demonstrated that M2b macrophage supernatant increased the proliferation, migration and tube formation of LECs, indicating that M2b macrophages may directly promote lymphangiogenesis. We also explored the mechanism by which M2b macrophages stimulate lymphangiogenesis. *In vivo*, the expression levels of VEGFC and VEGFR3 in the cardiac tissue of the M2b macrophage treatment group were significantly higher than those of the Vehicle treatment group. *In vitro*, the mRNA expression of VEGFC was upregulated in M2b macrophages, and the expression of VEGFR3 was upregulated in LECs treated with M2b macrophage supernatant. Therefore, we concluded that M2b macrophages promote lymphangiogenesis via the VEGFC/VEGFR3 pathway.

There are some limitations to this study. First, we focussed only on lymphatic counts, and did not examine whether there were changes in lymphatic structure or function. Second, the differences between the expressions of VEGFR3 and VEGFC in the Control group and the IR + Vehicle group were not statistically significant. There may be other mechanisms, such as the TGF-β/VEGFC pathway, that are activated to promote lymphangiogenesis after MI/RI (Kinashi et al. 2018). TGF-β is a key player in tissue fibrosis, and can promote VEGFC production leading to lymphangiogenesis and ‘fibrosis-related lymphangiogenesis’ in the development of renal and peritoneal fibrosis (Suzuki et al. 2012; Kinashi et al. 2013). We did not detect the expression of TGF-β or explore the mechanism of lymphangiogenesis related to MI/RI, but believe this will be of interest for future research. Third, the expression of VEGFC in M2b macrophages cannot be downregulated by siRNA, so we did not temporarily silence VEGFC to observe its importance for lymphangiogenesis and fibrosis.

Our study shows for the first time that M2b macrophages promote lymphangiogenesis to reduce myocardial fibrosis and improve heart function in the late stage of MI/RI. These effects may occur via the VEGFC/VEGFR3 pathway. This observation may be useful for development of myocardial protection therapies as it is now recognized that lymphangiogenic therapy is useful for the treatment of cardiovascular diseases.
